# Expression of Concern: Cytomegalovirus and Herpes Simplex Virus Effect on the Prognosis of Mechanically Ventilated Patients Suspected to Have Ventilator-Associated Pneumonia

**DOI:** 10.1371/journal.pone.0278002

**Published:** 2022-12-13

**Authors:** The PLOS ONE Editors

This article [1] has been identified as one of a series of submissions for which we have concerns about the reported research ethics approval information and the article’s adherence to PLOS research ethics policies.

PLOS will be investigating these concerns in accordance with COPE guidance and journal policies. Meanwhile, the *PLOS ONE* Editors issue this Expression of Concern.

## References

[1] Coisel Y, Bousbia S, Forel J-M, Hraiech S, Lascola B, Roch A, et al. (2012) Cytomegalovirus and Herpes Simplex Virus Effect on the Prognosis of Mechanically Ventilated Patients Suspected to Have Ventilator-Associated Pneumonia. PLoS ONE 7(12): e51340. 10.1371/journal.pone.0051340 23236477 PMC3517464

